# Initial spinal epidural hematoma symptoms mimic artery dissection

**DOI:** 10.1002/ccr3.8652

**Published:** 2024-03-08

**Authors:** Shunya Yabuki, Hiroyuki Hirai, Masayuki Miyajima, Takatomo Moro

**Affiliations:** ^1^ Department of 3rd Internal Medicine Shirakawa Kosei General Hospital Fukushima Japan; ^2^ Department of Radiology Shirakawa Kosei General Hospital Fukushima Japan; ^3^ Department of Orthopedic Surgery Shirakawa Kosei General Hospital Fukushima Japan

**Keywords:** acute upper‐back pain, artery dissection, elderly patient, hypertension, spontaneous spinal epidural hematoma

## Abstract

An elderly patient with upper back pain and hypertension was diagnosed as having spontaneous spinal epidural hematoma (SSEH) after excluding artery dissection. The initial symptoms of SSEH mimic those of artery dissection, and the symptoms of spinal damage frequently appear later. Physicians should, therefore, be mindful of SSEH.

## Graphical abstract

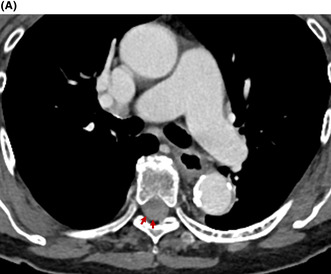

## 1 CLINICAL IMAGES

**FIGURE 1:**
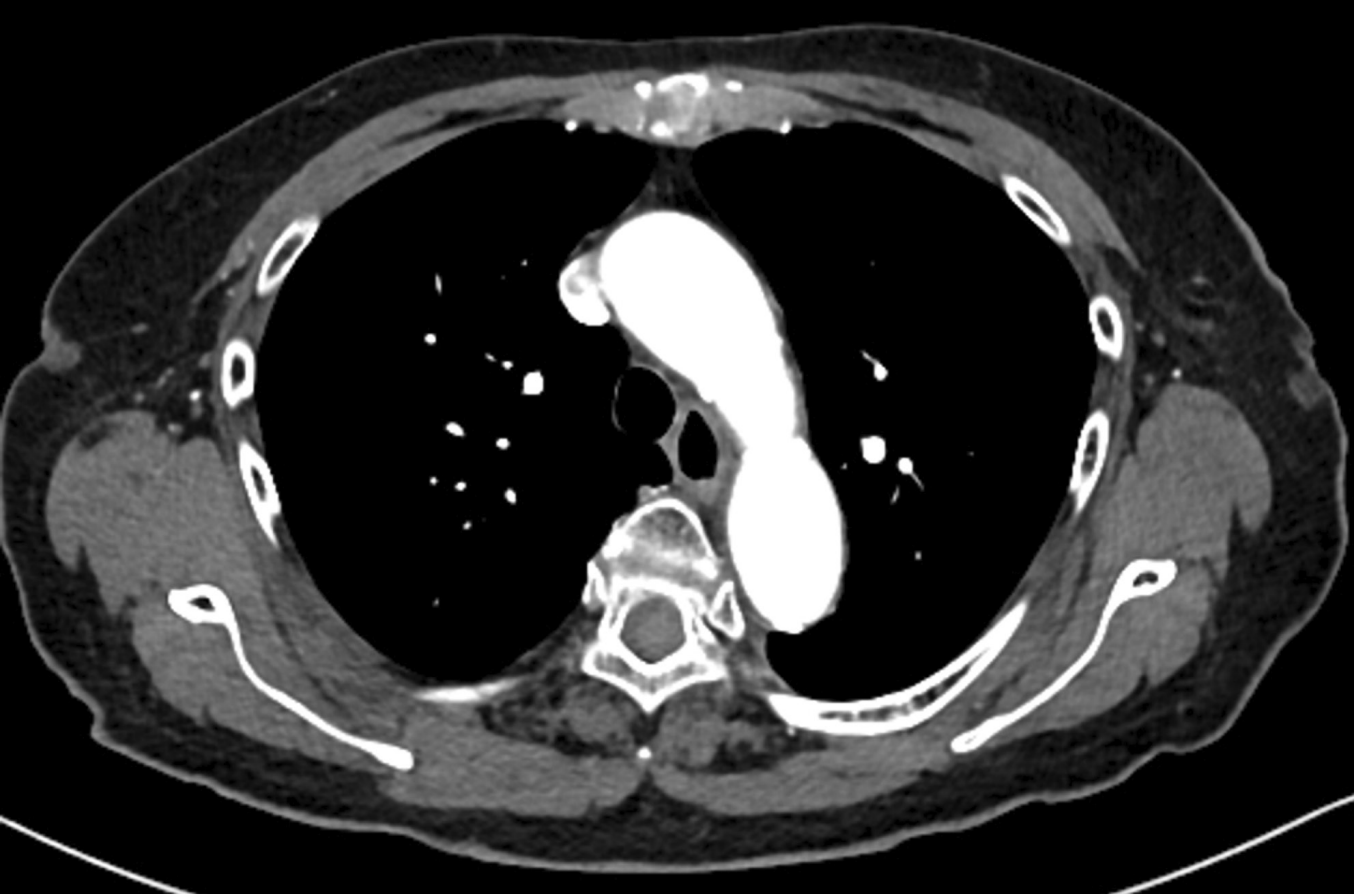
Contrast‐enhanced computed tomography. Contrast‐enhanced computed tomography show no artery dissection.

In September 2023, an 89‐year‐old woman presented with vomiting and upper back pain after evening. Although she was elderly, she independently performed activities of daily living with no signs of paralysis. The vital signs were recorded as follows; blood pressure, 186/106 mmHg; heart rate, 82 beats/min; temperature,36.9°C; spO_2_, 96% on room air. She was diagnosed with hypertension and dyslipidemia in her 70s. The patient was receiving daily doses of 5‐mg amlodipine, 2 mg‐candesartan, 30‐mg azosemide, 2.5‐mg Rosuvastatin, 1800‐mg eicosapentaenoic acid. She was not taking oral antiplatelet medications. The suspicion of artery dissection (AD) arose due to high blood pressure and the sudden onset of back pain. Contrast‐enhanced computed tomography (CT) revealed no evidence of AD (Figure 1). Laboratory examination revealed a slight increase in d‐dimer and brain natriuretic peptide (BNP) levels, indicative of normal hemostasis. The electrocardiogram showed no ischemic changes; therefore, ischemic heart disease and AD were excluded.

The above investigations ruled out other emergency diseases causing upper‐back pain such as pulmonary embolism, tension pneumothorax, esophageal rupture, and acute pancreatitis. The Manual Muscle Testing (MMT) score was 5/5, with no paralysis in the four limbs. After a few hours of monitoring, since there was no worsening of symptoms, we opted for careful observation.

However, the next morning, although upper back pain improved marginally, there was bilateral weakness in the lower limbs and the patient had difficulty walking, with a clear consciousness level and BP of 169/ 88 mmHg. She had no hemiplegia and dysarthria. Although the upper limb MMT score did not worsen, that of the lower limbs worsened to 4/5. Therefore, we suspected spinal cord injury at the thoracic vertebra level. The degree of disability of spinal cord injury was as follows: Frankel classification^1^: Grade C, and American Spinal Injury Association Impairment (ASIA) scale^2^: Grade D.

**FIGURE 2:**
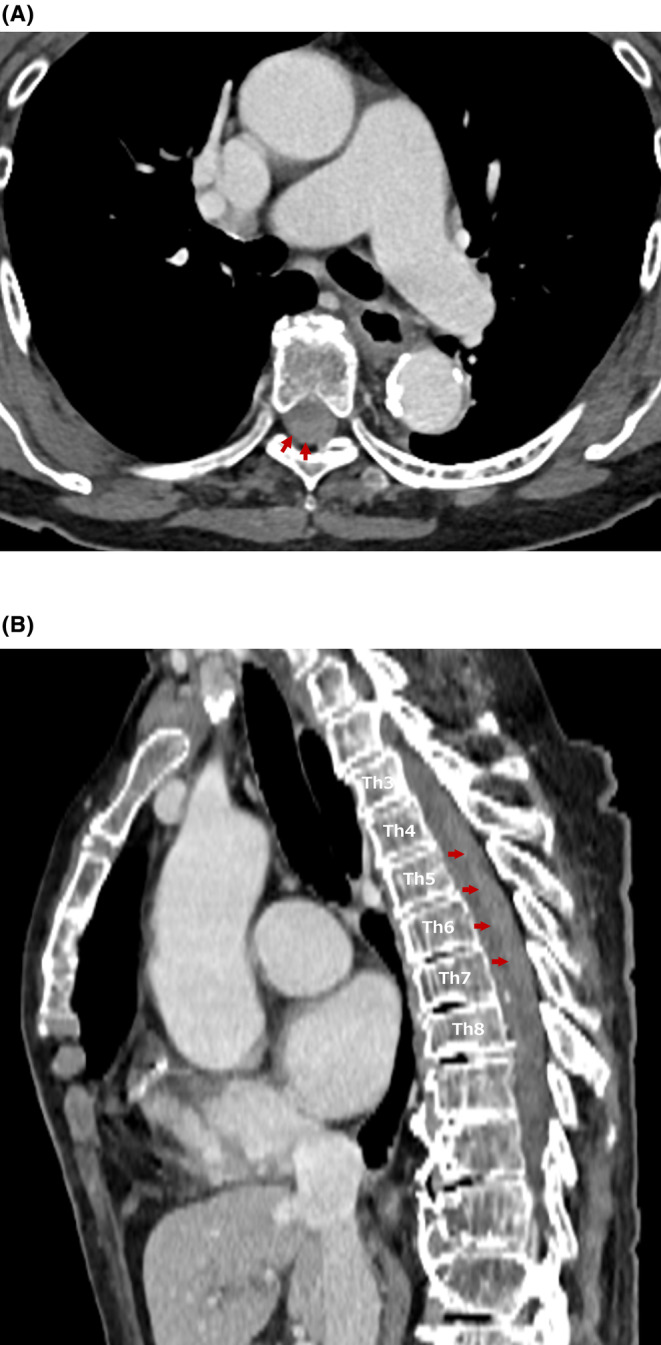
Axial and sagittal computed tomography. (A) Axial contrast‐enhanced computed tomography of the thoracic spine reveals a spinal epidural hematoma at six vertebral levels (red arrows). (B) Sagittal computed tomography reveals a spinal epidural hematoma from thoracic vertebra 2–7 (red arrows).

**FIGURE 3:**
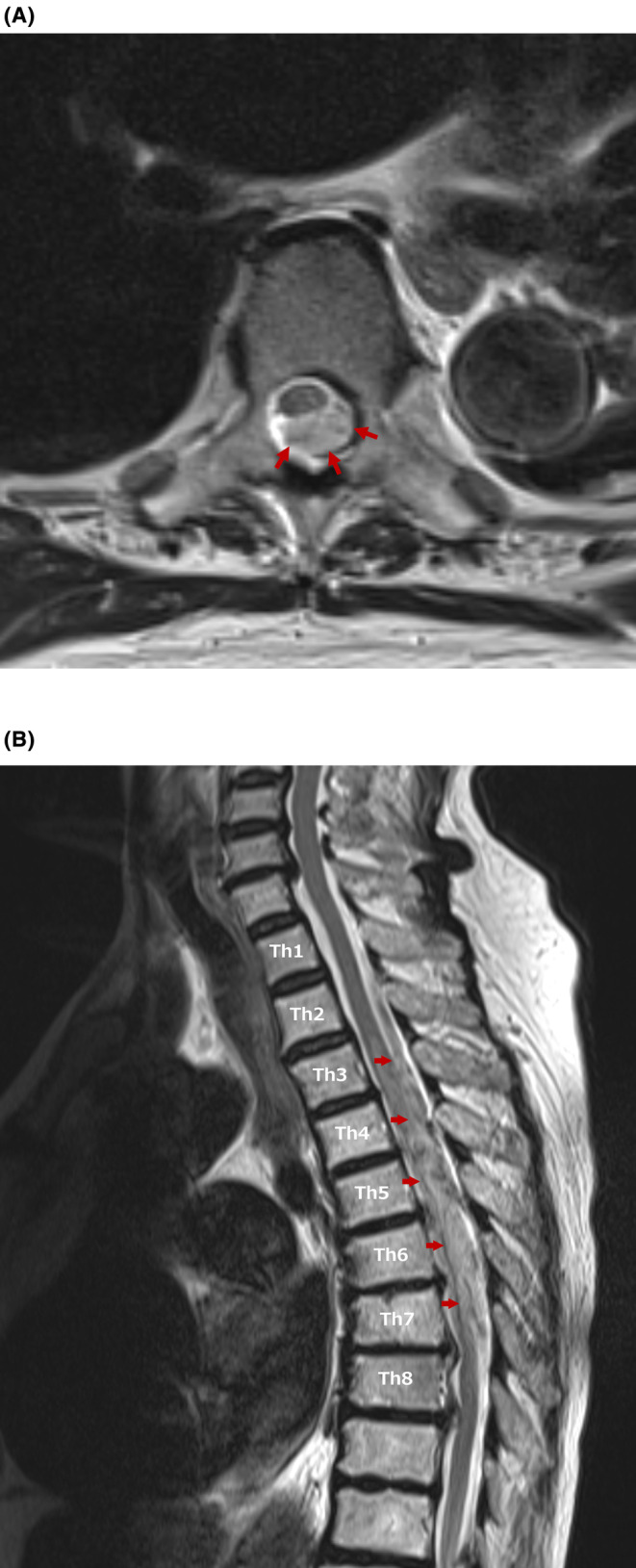
Axial and sagittal magnetic resonance imaging. (A) Axial magnetic resonance imaging of the thoracic spine reveals a spinal epidural hematoma at six thoracic vertebral levels (red arrows). (B) Sagittal magnetic resonance imaging reveals a spinal epidural hematoma from thoracic vertebra 2–7 (red arrows).

When we reconstructed the CT with sagittal imaging, we found epidural hematoma in the spinal cord at th2‐6 (Figure 2A,B). Spinal cord MRI was performed (Figure 3A,B); Such as spinal cord infarction, transverse myelitis, malformation of spinal artery, and herniated disc were excluded. Therefore, she was diagnosed with spontaneous spinal epidural hematoma (SSEH). The patient was transferred to an advanced medical institution.

Since the symptoms improved slightly following transfer, and her family did not want an invasive intervention, she was kept under observation. The symptoms improved within a few days and spin MRI revealed disappearance of the hematoma.

## 2 DISCUSSION

SSEH is a rare condition characterized by sudden back pain and spinal cord symptoms resulting from the formation of an epidural hematoma without any associated trauma or underlying medical causes.^1^, ^3^ Despite the low incidence rate of 0.1 in 10 million,^1^, ^3^ SSEH is considered an urgent condition, and an early diagnosis is crucial. However, due to the diverse symptoms that often mimic those of cerebral infarction, accurate diagnosis poses a significant challenge. Furthermore, during the early stages of the disease, upper back pain is the sole symptom, and paralysis typically develops later, adding complexity to clinical decision‐making.^1^, ^3^ Therefore, we present an instructive case of a patient experiencing upper back pain diagnosed with SSEH after ruling out AD.

Al‐Mutair et al. indicated mean time of 3 h between onset of pain at the location of hemorrhage and full clinical presentation.^4^ In this case, the occurrence of spine injury symptoms from onset of upper back pain were approximately 18 h, which made the diagnosis difficult. The reported mortality rate is 5.7%,^5^ which is not low, and the initial diagnosis is crucial. In this case, the diagnosis could have been made sooner if the hematoma had been detected on the first axial CT scan. Thus, this case implies that clinicians should be aware of SSEH at the time of diagnosis for acute upper‐back pain, prior to the onset of symptoms associated with spine injuries.

Although SSEH commonly occurs during 60s and 70s,^3^ elderly patients aged ≥80 may be affected. SSEH is also associated with hypertension^3^ and use of antiplatelet agents,^3^, ^6^ and our patient had hypertension. Unfortunately, the cause of SSEH could not be identified in our patient as the spinal epidural venous plexus is a common source of hemorrhage, and that the vascular wall is fragile, lacking venous valves, which is easily disrupted by stresses such as elevated blood pressure.^3^

Treatment of SSEH is controversial, particularly when choosing between conservative or surgical treatment.^1^, ^3^, ^4^, ^5^ Fortunately, this case was resolved with cautious observation alone—invasive surgery was not required. The reasons might be as follows: (1) ASIA score D and mild neurological impairments, and (2) no use of antiplatelet agents. However, since not every case might improve with diligent observation alone, it is vital to gather evidence regarding the treatment approach and time.

In summary, SSEH should be considered in elderly patients with acute upper back pain and hypertension. The initial symptoms mimic those of AD, physicians must not overlook SSEH.

## AUTHOR CONTRIBUTIONS

**Shunya Yabuki:** Conceptualization; data curation; formal analysis; investigation; methodology; writing – review and editing. **Hiroyuki Hirai:** Conceptualization; data curation; formal analysis; investigation; methodology; project administration; writing – original draft; writing – review and editing. **Masayuki Miyajima:** Conceptualization; data curation; formal analysis; methodology; writing – review and editing. **Takatomo Moro:** Conceptualization; data curation; formal analysis; methodology; writing – review and editing.

## FUNDING INFORMATION

No funding was received for conducting this study.

## CONFLICT OF INTEREST STATEMENT

The authors state that they have no conflict of interest.

## CONSENT STATEMENT

Written informed consent was obtained from the patient to publish this report in accordance with the journal's patient consent policy.

## Data Availability

The data that support the findings of this study are available from the corresponding author upon reasonable request.
